# Immunotherapeutic efficacy of recombinant canine IL-15 as an adjunct to chemotherapy in canine lymphoma

**DOI:** 10.3389/fvets.2025.1596084

**Published:** 2025-05-30

**Authors:** Min-Hee Kang, Jaeil Lee, Sang-Ki Kim, Kyeyoung Koh, Mi-Ae Kang, Hee-Myung Park

**Affiliations:** ^1^Department of Bio-Animal Health, Jangan University, Hwaseong, Republic of Korea; ^2^Biomaterial R&D Center, VaxCell-Bio, Gwangju, Republic of Korea; ^3^Vet&Gene, Seongnam, Republic of Korea; ^4^Department of Veterinary Internal Medicine, College of Veterinary Medicine, Konkuk University, Seoul, Republic of Korea

**Keywords:** tumor biomarkers, canine lymphoma, recombinant canine interleukin-15, immunotherapy, quality of life

## Abstract

**Introduction:**

Canine lymphoma is a common hematopoietic malignancy with variable response to standard chemotherapy. Interleukin-15 (IL-15) is known to enhance cytotoxic lymphocyte activity, and this study aimed to evaluate the efficacy and safety of a recombinant canine IL-15 (rcIL-15) as an adjunct to chemotherapy in dogs with lymphoma.

**Methods:**

A total of 61 dogs diagnosed with lymphoma were enrolled in a 12-week clinical study. The test group received rcIL-15 in combination with standard chemotherapy, while the control group received chemotherapy alone. Outcome measures included tumor response rates, tumor biomarker levels (TK-1, LDH, β2-microglobulin), quality of life (QOL) assessments, and adverse event monitoring.

**Results:**

Of the 61 dogs enrolled, 37 completed the study. The test group demonstrated a higher overall response rate (complete + partial response: 77.8%) compared to the control group (57.9%). Disease progression was observed in 16.7% of dogs in the test group versus 31.6% in controls. Tumor biomarkers were significantly reduced in the test group: TK-1 at 8 weeks (*p* < 0.0001), LDH at 12 weeks (*p* = 0.005), and β2-microglobulin at both 8 and 12 weeks (*p* < 0.05). IFN-γ levels remained stable. QOL parameters, including appetite, activity, and happiness, showed significant improvement. Adverse events were mild, mostly gastrointestinal, and manageable.

**Discussion:**

Adjunctive rcIL-15 therapy improved tumor response, reduced biomarker levels, and enhanced QOL with an acceptable safety profile. These findings support the potential of rcIL-15 as a safe and effective immunotherapeutic adjunct for canine lymphoma, meriting further investigation in larger-scale trials.

## 1 Introduction

The extended lifespan of companion animals has contributed to a rise in age-related diseases, including cancer, diabetes, obesity, cardiovascular disorders, and immune-mediated conditions (1). Among these, cancer poses one of the most significant health challenges for aging dogs, with an incidence rate substantially higher than in humans. Studies suggest that cancer is a leading cause of death in dogs over 8 years old. In the United States, cancer accounts for a large proportion of mortality in adult dogs (2, 3). Despite this high prevalence, veterinary-specific anticancer drugs remain limited, and human chemotherapeutics are often repurposed for use in dogs, raising concerns about their safety, efficacy, and cost-effectiveness (4).

Lymphoma is one of the most common malignant cancers in dogs, underscoring the critical importance of early detection and effective treatment (5, 6). Although chemotherapy, particularly CHOP protocols, is the current standard for managing canine lymphoma, its limitations are well-documented. These include a high relapse rate, incomplete tumor control, and significant side effects that can diminish quality of life (4). Addressing these challenges necessitates the exploration of innovative therapeutic strategies. Immunotherapy has emerged as a promising approach, leveraging the body’s immune system to enhance tumor control and improve patient outcomes (7).

Among various immunotherapeutic agents, interleukin-15 (IL-15) has shown substantial promise in human oncology (8). IL-15 activates critical immune cells, including natural killer (NK) cells and cytotoxic T cells, thereby enhancing tumor cell lysis and supporting the long-term survival of memory T cells (9, 10). Unlike interleukin-2 (IL-2), IL-15 avoids stimulating regulatory T cells (Tregs), which can suppress anti-tumor immunity, and is associated with lower toxicity and fewer side effects (11, 12). These properties make IL-15 an ideal candidate for combination therapies designed to overcome tumor immune evasion and improve therapeutic outcomes.

The development of species-specific immunotherapies in veterinary medicine has gained increasing attention, emphasizing the need to address the unique physiological and immunological characteristics of companion animals (13). Advances in microbial expression systems have enabled the cost-effective production of recombinant biologics, offering scalable solutions for innovative treatments.

This study evaluates the safety, efficacy, and immune-modulating effects of a newly developed rcIL-15 when combined with standard chemotherapy in dogs diagnosed with lymphoma. By investigating tumor response, quality of life, and biomarker changes, this clinical research aims to provide insights into the potential of IL-15-based immunotherapy to enhance existing treatment protocols in veterinary oncology.

## 2 Materials and methods

### 2.1 Study population and eligibility criteria

This clinical study was conducted with the approval of the Institutional Animal Care and Use Committee (Approval Number: CPT-23-001-R). Dogs with lymphoma were recruited from 23 veterinary hospitals in South Korea from January 2023 to August 2024. A total of 61 client-owned dogs were enrolled based on inclusion criteria specified in the clinical trial protocol. All dogs were newly diagnosed and treatment-naïve, with no prior exposure to chemotherapy or immunotherapy before enrollment. All cases underwent evaluation using a standardized diagnostic protocol, which included cytological or histopathological confirmation and clinical staging to ensure diagnostic consistency. Lymphoma subtypes were primarily classified based on cytological evaluation of lymph node aspirates. Immunophenotyping was performed when sufficient sample quality and volume allowed; however, due to limitations, not all cases underwent immunophenotyping, and precise classification into B-cell or T-cell lymphoma was not available for all subjects. Written informed consent was obtained from all owners prior to enrollment, and all procedures adhered to ethical guidelines.

Eligible dogs were those without significant comorbidities that could interfere with the evaluation of the study outcomes. Dogs with pre-existing conditions were included if these conditions were considered unlikely to affect the assessment of anti-cancer effects, based on the attending veterinarian’s professional judgment. Final eligibility decisions were reviewed by the study’s advisory board to ensure consistency and reliability.

Dogs were excluded from the study if they had other malignant tumors unrelated to lymphoma, as these could confound study outcomes. Additional exclusion criteria included severe health deterioration or critical illnesses, inability to undergo standard lymphoma treatments such as CHOP-based chemotherapy, or significant underlying conditions causing immunosuppression or impairing treatment efficacy. Dogs currently enrolled in other clinical trials, as well as those that were pregnant or lactating, were also excluded. In cases of uncertainty, eligibility was determined by the attending veterinarian, with final approval reviewed by the advisory board.

### 2.2 Study protocol

This clinical trial evaluated the efficacy and safety of rcIL-15 in combination with standard chemotherapy in dogs diagnosed with lymphoma. All enrolled dogs were randomly assigned to either the control group, receiving standard chemotherapy alone, or the test group, receiving standard chemotherapy combined with rcIL-15, using simple randomization. Standard chemotherapy consisted of a modified CHOP protocol, including doxorubicin, cyclophosphamide, vincristine, and prednisone, administered according to a standardized schedule adapted for canine lymphoma. The study was conducted as an open-label trial; however, to minimize assessment bias, veterinarians evaluating clinical outcomes, including lymph node size, were blinded to group allocation. The study spanned 3 months, during which clinical outcomes and laboratory parameters were systematically assessed at five scheduled visits: baseline (pre-treatment), and at weeks 2, 4, 8, and 12.

This study utilized a commercially developed rcIL-15 (VaxCell Biotherapeutics Co., Ltd., Hwasun, South Korea). RcIL-15 was generated using *E. coli* BL21(DE3) as the host and pET30a(+) as the expression vector, following previously described methods (14). The protein was formulated as a clear injectable solution and supplied in 1 mL glass vials. A dosage of 20 μg/kg/day was selected based on prior optimization studies in healthy dogs, which demonstrated effective stimulation of immune effector cells without significant adverse effects (14, 15). RcIL-15 was administered by intravenous infusion over two cycles, each consisting of four consecutive days of daily infusion followed by a 10-day rest period. The drug was diluted in ≤10 mL of normal saline and infused over 10 min through an indwelling IV catheter. Vials were stored at 2–8°C to maintain stability and ensure proper handling throughout the study.

### 2.3 Clinical assessments

Comprehensive clinical assessments were conducted during each scheduled visit. General observations included a review of each dog’s medication history, documenting all drugs administered within 2 weeks preceding the first visit. Medical history was obtained to identify recent illnesses, surgical procedures, or clinical signs observed within the past 3 months. Physical examinations were performed during each visit, measuring vital signs such as body weight, body condition score (BCS, using a 1–5 scale), rectal temperature, heart rate, respiratory rate, and systolic blood pressure.

Owner-reported QoL was assessed using validated questionnaires covering appetite, activity, hydration, and overall well-being (16). Although owners were aware of their dogs’ treatment group assignments due to the open-label design, the use of standardized and validated questionnaires helped minimize subjectivity and improve the consistency of QoL evaluations.

### 2.4 Tumor biomarker analysis

Serum samples were collected at all scheduled visits from dogs diagnosed with lymphoma after obtaining informed consent from their owners. The samples were immediately stored at −20°C until analysis. Thymidine kinase 1 (TK1), interferon-gamma (IFN-γ), β2-microglobulin (β2M), and lactate dehydrogenase (LDH), which are associated with tumor activity and immune response, were measured using standardized protocols. TK1 levels were quantified using the OKIA00205 ELISA kit (Aviva Systems Biology, San Diego, California, United States), IFN-γ levels were measured using a commercial ELISA kit (MBS453504; MyBioSource, San Diego, California, United States), and β2M concentrations were assessed using the MBS703912 ELISA kit (MyBioSource). ELISA measurements were performed using the SpectraMax ABS Plus microplate reader (Molecular Devices, San Jose, California, United States) at 450 nm with 630 nm as the reference wavelength. The three assays showed high accuracy determined by linearity un-der dilution (coefficient of correlation close to 1). LDH levels were analyzed using the DRI-CHEM NX500i biochemical analyzer (FUJI Corporation, Osaka, Japan) following the manufacturer’s instructions. Relative changes in biomarker levels were normalized against baseline (0 weeks) values to assess trends over time.

### 2.5 Safety assessments

Hematological, biochemical, and electrolyte parameters were monitored at all scheduled visits to evaluate the safety profile of rcIL-15. Complete blood count (CBC) tests included measurements of white blood cell count, red blood cell count, hemoglobin, hematocrit, and platelet count. Serum biochemistry included assessments of liver and kidney function markers such as ALT, ALKP, creatinine, and BUN. Electrolytes, including sodium, potassium, and chloride, were also analyzed.

Adverse events were recorded and categorized based on severity using the Veterinary Cooperative Oncology Group-Common Terminology Criteria for Adverse Events version 2 (VCOG-CTCAE v2) (17). Vital signs, body weight, temperature, and clinical symptoms such as diarrhea were systematically recorded and analyzed. Potential as-sociations with rcIL-15 administration were evaluated by the attending veterinarian.

### 2.6 Statistical analysis

Data were expressed as mean ± standard deviation (SD) or mean ± standard error (SE). Normality was assessed using the Kolmogorov–Smirnov test. Depending on the results, comparisons between the test and control groups were conducted using either Student’s *t*-test (for normally distributed data) or the Mann–Whitney *U* test (for non-normal data). For categorical variables, Pearson’s chi-square test was used, while Fisher’s exact test was applied when sample sizes were small. Repeated measures ANOVA were used to evaluate time, group, and time-by-group interaction effects. *Post hoc* comparisons following repeated-measures ANOVA were adjusted using the Bonferroni correction to control for multiple testing. For non-parametric data, analyses were performed using the nparLD package in R. As this study was designed as an exploratory clinical trial, a formal sample size calculation (power analysis) was not performed. The number of enrolled dogs was determined based on feasibility and the preliminary nature of the investigation.

Statistical significance was set at *p* < 0.05. All analyses were conducted using SPSS version 22 (SPSS Inc., Chicago, IL, United States), GraphPad Prism version 9.5.1 (GraphPad Software Inc., Boston, MA, United States), and R version 4.4.1.

## 3 Results

### 3.1 Characteristics of enrolled dogs

Of the 61 dogs enrolled, 37 completed the study and were analyzed (test group: 18, control: 19). Table 1 summarizes the base-line characteristics of the enrolled dogs. The test and control groups were comparable in age, weight, sex, neuter status, and breed distribution (*p* > 0.05), indicating no significant baseline differences between the groups. Most dogs were small to medium sized breeds and aged over 9 years.

**Table 1:** Baseline characteristics of enrolled dogs in the lymphoma clinical trial.

Characteristic	Test group (*n* = 18)	Control group (*n* = 19)	*p*
Age (years)	9.2 ± 3.7	9.5 ± 3.7	0.795
Weight (kg)	7.5 ± 4.2	10.7 ± 11.1	0.843
Sex (*n*)			
Male	1	2	0.687
Castrated Male	11	12	
Female	0	1	
Spayed Female	6	4	
Breed (*n*)			
Maltese	6	4	
Mixed	2	3	
Shih Tzu	2	1	
Cocker Spaniel	2	1	
Welsh Corgi	2		
Pomeranian	1		
Italian Greyhound	1		
Dachshund	1		
French Bulldog	1		
Poodle		3	
Golden Retriever		2	
Others^a^			

Age and weight values are presented as mean ± standard deviation.

a Others: Yorkshire Terrier, Bichon Frise, Miniature Schnauzer, Labrador Retriever, Shetland Sheepdog.

The tumor stages of the enrolled dogs ranged from Stage 1 to 5, with a majority classified as Stage 3 or 4 (Table 2). No significant differences in tumor stage distribution were observed between the test and control groups (*p* = 0.477), ensuring balanced disease severity across the cohorts.

**Table 2:** Tumor staging information of enrolled dogs in the lymphoma clinical trial.

Tumor stage^a^	Test group (*n* = 18)	Control group (*n* = 19)	*p*
Stage 1	1	3	0.477
Stage 2	2	1	
Stage 3	6	3	
Stage 4	6	10	
Stage 5	3	2	

a Stage 1—single lymph node enlarged, Stage 2—multiple nodes enlarged on either the front or back half of the body, Stage 3—multiple nodes enlarged on both front and back halves of the body, Stage 4—involvement of the liver and/or spleen, Stage5—bone marrow involvement or involvement of other organs (e.g., gastrointestinal, skin, nervous system).

### 3.2 Vital parameters during the study period

Six vital parameters, including body weight, body condition score (BCS), rectal temperature, heart rate, respiratory rate, and blood pressure, were monitored across five time points (pre-treatment, 2 weeks, 4 weeks, 8 weeks, and 12 weeks). No significant differences in time, group, or time-group interactions were observed for body weight, BCS, temperature, heart rate, or respiratory rate during the 12-week evaluation period (*p* > 0.05; Table 3).

**Table 3:** Variations in vital parameters between the lymphoma test group and the control group.

Vital parameters	Groups	0 W	2 W	4 W	8 W	12 W	*p* (time)	*p* (group)	*p* (time^a^ group)
Body weight (kg)	Test	7.5 ± 4.2	7.4 ± 4.2	7.6 ± 4.4	7.5 ± 4.3	7.6 ± 4.3	0.584	0.789	0.728
	Control	10.7 ± 11.1	10.9 ± 11.5	11.1 ± 11.7	11.1 ± 11.7	11.3 ± 12.1			
BCS (5 scale)	Test	3.1 ± 0.5	3.2 ± 0.4	3.2 ± 0.4	3.2 ± 0.4	3.3 ± 0.5	0.700	0.565	0.074
	Control	3.3 ± 0.6	3.3 ± 0.6	3.3 ± 0.6	3.3 ± 0.5	3.2 ± 0.6			
Temp. (°C)	Test	38.8 ± 0.5	38.8 ± 0.4	38.7 ± 0.4	38.8 ± 0.5	38.8 ± 0.5	0.894	0.914	0.122
	Control	38.6 ± 0.4	38.6 ± 0.5	38.8 ± 0.4	38.6 ± 0.5	38.6 ± 0.7			
HR (bpm)	Test	134.9 ± 26.2	138.6 ± 21.0	134.7 ± 15.7	135.3 ± 22.3	138.9 ± 19.5	0.253	0.858	0.634
	Control	135.6 ± 25.9	130.7 ± 29.6	131.0 ± 30.2	133.2 ± 25.7	134.6 ± 24.8			
RR (/min)	Test	32.0 ± 14.3	27.7 ± 10.2	27.5 ± 10.5	28.9 ± 12.7	26.4 ± 9.2	0.989	0.910	0.061
	Control	23.9 ± 6.8	27.6 ± 10.6	27.8 ± 9.0	27.4 ± 7.6	29.5 ± 10.3			
BP (mmHg)	Test	137.3 ± 18.3	139.3 ± 9.9	129.6 ± 13.8	132.4 ± 12.2	134.3 ± 18.4	0.006^a^	0.188	0.765
	Control	142.4 ± 17.8	148.5 ± 24.1	138.1 ± 17.0	138.4 ± 22.9	134.7 ± 17.4			

Values are presented as mean ± standard deviation.

0 W: Before administration of rcIL-15; 2 W, 4 W, 8 W, 12 W: 2, 4, 8, and 12 weeks after the first administration of rcIL-15, respectively.

BCS, body condition score evaluated on a 5-point scale; Temp., temperature; HR, heart rate; RR, respiratory rate; BP, blood pressure.

a Statistical significance: *p* < 0.05.

Blood pressure showed significant variation over time (*p* = 0.006) but no differences between groups or time-group interactions (*p* > 0.05). While minor reductions in blood pressure were observed over time in both groups, the values remained within normal physiological ranges, with no clinical significance.

### 3.3 Lymph node size reduction

The therapeutic efficacy of rcIL-15 was evaluated using lymph node size changes categorized into CR (complete remission), PR (partial response), SD (stable disease), and PD (progressive disease). As shown in Table 4, the combined rate of CR and PR was higher in the test group (77.8%) compared to the control group (57.9%), though the difference was not statistically significant (*p* = 0.375). Similarly, the proportion of dogs with disease progression (PD) was lower in the test group (16.7%) than in the control group (31.6%), suggesting a trend toward better outcomes in the test group.

**Table 4:** Changes in lymph node size between test and control groups in the lymphoma clinical trial.

Lymph node size	Test group (*n* = 18)	Control group (*n* = 19)	*p*
CR, *n* (%)	10 (55.6)	10 (52.6)	0.375
PR, *n* (%)	4 (22.2)	1 (5.3)	
SD, *n* (%)	1 (5.6)	2 (10.5)	
PD, *n* (%)	3 (16.7)	6 (31.6)	

CR, complete response; PR, partial response; SD, stable disease; PD, progressive disease.

### 3.4 Tumor biomarkers

**Figure 1:**
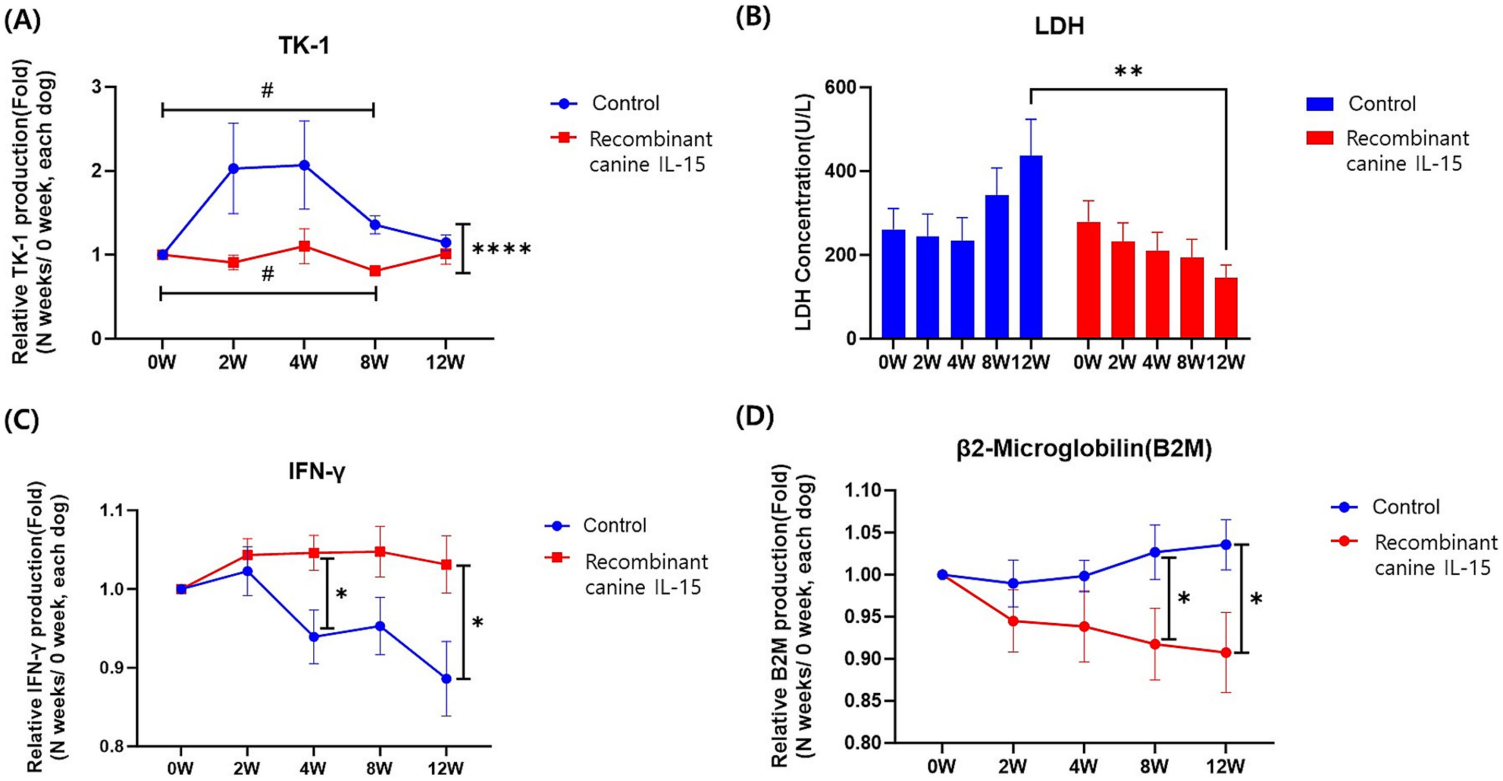
Changes in serum tumor biomarkers in dogs with lymphoma treated with a recombinant canine IL-15. Panels includes **(A)** TK-1, **(B)** LDH, **(C)** IFN-γ and **(D)** β2M. Panels illustrate the changes in relative serum levels of key biomarkers over time in the test group (red) and control group (blue). Measurements were taken before treatment (0 W) and at 2, 4, 8, and 12 weeks post-treatment initiation. Data are expressed as relative concentrations normalized to pre-treatment levels (0 W) and presented as mean ± SEM. Statistical significance is indicated as follows: #, *; *p* < 0.05; ##, **; *p* < 0.01; ###, ***; *p* < 0.001; ####, ****; *p* < 0.0001. The symbol # represents significance within groups, while * indicates significance between groups.

To evaluate the clinical effectiveness of rcIL-15, the concentration of tumor-related biomarkers, including TK-1, LDH, IFN-γ, and β2M was measured using serum samples and the results are presented in Figure 1.

#### 3.4.1 TK-1

TK-1 levels in both the control and test groups were analyzed at different time points during the study (Figure 1A). At baseline (0 weeks), TK-1 concentrations were not significantly different between the two groups (test group: 5.24 ± 8.95 ng/mL; control group: 3.97 ± 5.80 ng/mL, *p* > 0.05). By 8 weeks, TK-1 was significantly lower in the test group (*p* < 0.0001), reflecting tumor suppression. Within-group comparisons revealed significant reductions in the test group from baseline to 8 weeks (*p* = 0.0148), while the control group displayed a relative increase (*p* = 0.0138) during this period. The reduction in TK-1 levels over time suggests that rcIL-15 may contribute to tumor suppression.

#### 3.4.2 LDH

LDH concentrations in the control and test groups were measured at multiple time points throughout the study (Figure 1B). At baseline (0 weeks), LDH concentrations were comparable between the control group (261.30 ± 266.70 U/L) and the test group (279.10 ± 277.00 U/L, *p* = 0.803). Over time, the control group demonstrated a progressive increase in LDH levels, while the test group showed a decreasing trend. Although these trends within each group were not statistically significant (*p* > 0.05), at 12 weeks, LDH levels in the test group (145.60 ± 126.40 U/L) were significantly lower than those in the control group (436.60 ± 382.40 U/L, *p* = 0.005). These findings highlight the potential of rcIL-15 to mitigate increases in LDH levels, which are often associated with tumor burden and cellular damage.

#### 3.4.3 IFN-γ

IFN-γ levels were analyzed throughout the study (Figure 1C). At base-line (0 weeks), the mean IFN-γ concentrations were not significantly different between the control group (69.06 ± 15.96 pg/mL) and the test group (63.32 ± 10.93 pg/mL, *p* > 0.05). The control group showed a steady decline in the relative IFN-γ concentration ratio, with a decline observed between 4 weeks (0.94 ± 0.16 pg/mL) and 12 weeks (0.89 ± 0.21 pg/mL). In the test group, IFN-γ levels remained relatively stable, with no statistically significant changes within the group across the study period (*p* > 0.05). However, between-group comparisons revealed that the relative IFN-γ concentration ratios were consistently higher in the test group compared to the control group, particularly at 4 weeks (*p* = 0.012) and 12 weeks (*p* = 0.020). The relatively stable IFN-γ levels observed in the test group may reflect the immune-modulatory effects of rcIL-15 compared to the control group.

#### 3.4.4 β2M

Relative concentration ratios of β2M were analyzed over the study period (Figure 1D). At baseline (0 weeks), β2M levels were similar between the control group (0.28 ± 0.11 μg/mL) and the test group (0.32 ± 0.18 μg/mL, *p* = 0.341). Over time, both groups exhibited stable β2M levels with no significant intragroup changes (*p* > 0.05). However, by 8 and 12 weeks, the control group showed significantly higher relative concentration ratios compared to the test group (8 weeks: control 1.03 ± 0.15 vs. test 0.92 ± 0.19, *p* = 0.048; 12 weeks: control 1.04 ± 0.13 vs. test 0.91 ± 0.19, *p* = 0.031). Elevated β2M levels are associated with poor prognosis in lymphoma, and the observed lower β2M ratios in the test group may indicate a beneficial therapeutic effect.

### 3.5 Owner quality of life assessments

Owner-reported quality of life evaluations demonstrated improvements in dogs treated with rcIL-15 compared to controls. Significant improvements were reported in happiness (*p* = 0.024), appetite (*p* = 0.040), and mobility (*p* = 0.012), reflecting the positive impact of rcIL-15 on emotional well-being and physical activity (Supplementary Table 1).

### 3.6 Safety and side effects

Safety assessments, including hematological, biochemical, and electrolyte analyses, showed no significant abnormalities in either group throughout the study period. The majority of side effects were mild gastrointestinal symptoms, such as diarrhea, vomiting, and reduced appetite. These events were transient and resolved without intervention, except for two cases of vomiting that were considered potentially related to rcIL-15 administration. Both cases were managed with supportive care, and no further complications were observed.

## 4 Discussion

This study evaluated the efficacy and safety of a newly developed rcIL-15 as an adjunct to standard chemotherapy in dogs with lymphoma, focusing on its impact on clinical outcomes, tumor biomarkers, and quality of life. The findings suggest that rcIL-15 offers potential therapeutic benefits when combined with standard chemo-therapy.

Canine lymphoma is one of the most common malignancies in dogs, and chemotherapy is widely regarded as the standard of care (5, 6). However, response rates to chemotherapy vary, with complete remission typically achieved in 60–90% of cases, but relapse occurring in most patients within 6–12 months (6). The addition of immunotherapeutic agents like rcIL-15 represents a novel approach aimed at addressing these limitations by enhancing tumor immune surveillance and reducing relapse rates (8). Although the dosage of 20 μg/kg/day employed in this study was higher than that reported in human IL-15 trials and other canine studies, it was based on prior preclinical evaluations in dogs that demonstrated good tolerability and effective immune activation (14, 15). Species-specific differences, including pharmacokinetics and immune responsiveness, may account for the variance in optimal dosing. Importantly, no severe adverse events related to rcIL-15 administration were observed in this study, supporting the safety of this dosing regimen in canine patients. This study’s findings support the immunological mechanisms previously attributed to IL-15. Preservation of IFN-γ levels in the test group suggests sustained NK and T-cell activity (9, 10, 18). Reductions in TK-1, a marker of tumor proliferation (19), and LDH, associated with hypoxia and tumor burden (20), further indicate the potential of rcIL-15 to modulate the tumor microenvironment. These biomarker changes align with the known effects of IL-15 on enhancing tumor suppression through both immune activation and metabolic pathways, potentially improving treatment outcomes (21–23).

The maintenance of IFN-γ levels in the test group compared to their decline in the control group underscores the immunomodulatory effects of rcIL-15. IL-15 has been shown to promote the survival and proliferation of memory T cells and NK cells while avoiding the immunosuppressive effects associated with IL-2, such as regulatory T-cell expansion (9–12, 24). This study provides preliminary evidence that these immunological mechanisms may also apply to canine lymphoma, supporting further exploration of IL-15 as an adjunctive therapy in veterinary oncology. Additionally, reductions in β2M in the test group highlight its potential role in modulating immune and tumor interactions. Elevated β2M levels are associated with poor prognosis in both humans and dogs with lymphoma (25, 26) making its reduction a clinically relevant finding.

The combination of rcIL-15 and standard chemotherapy demonstrated higher response rates (CR and PR) in the test group compared to the control group. While statistical significance was not reached, the reduced rate of PD in the test group (16.7% vs. 31.6%) suggests that rcIL-15 may provide an additional benefit in mitigating disease advancement. Moreover, tumor biomarkers such as TK-1, LDH, IFN-γ, and β2M showed trends that favor the test group, indicating potential immunomodulatory and tumor-suppressive effects.

This study underscores the importance of owner-reported quality of life, an often overlooked aspect of veterinary oncology. The test group exhibited notable improvements in happiness, mental health, appetite, hygiene, hydration status, and activity levels, highlighting the broader benefits of rcIL-15 beyond tumor control. Improvement in these quality of life parameters indicates that rcIL-15 may help alleviate some of the side effects of chemotherapy, particularly those related to patient well-being, which are common in both human and veterinary oncology patients (4). Additionally, the reduced frequency of clinical symptoms in the test group supports its potential role in improving overall patient well-being during treatment.

This study has several limitations that should be considered when interpreting the findings. One limitation is the relatively small sample size, which may have limited the statistical power to detect significant differences in some clinical endpoints, particularly in response rates. A formal *a priori* power analysis was not performed, and sample size determination was based on preliminary feasibility considerations. Although favorable trends were observed in biomarker changes and clinical outcomes, larger studies are necessary to validate these findings. Another limitation is the short follow-up period of 12 weeks, which precluded assessment of long-term outcomes such as progression-free survival and overall survival. The 12-week timeframe was initially selected to capture early biomarker changes, initial tumor responses, and safety signals; however, extended follow-up is necessary in future studies to better evaluate the durability of clinical benefits.

Additionally, although lymphoma subtypes were classified based on cytological evaluation in all cases, immunophenotyping was not consistently performed due to limitations in sample quality and availability. As a result, precise classification into B-cell or T-cell lymphoma was not possible in all subjects, which may have influenced treatment response interpretation. Furthermore, direct immune cell profiling, such as flow cytometric analysis of lymphocyte subsets, was not conducted. The evaluation of rcIL-15’s immunomodulatory effects in this study relied primarily on indirect tumor biomarkers and clinical outcomes. Future investigations incorporating immune cell profiling would provide a more comprehensive understanding of rcIL-15’s biological effects. In addition, while differences in tumor biomarkers (TK-1, LDH, β2M) were observed between groups, formal correlation analyses between biomarker changes and objective clinical responses (such as lymph node size reduction) were not performed. Establishing these correlations would strengthen the prognostic and predictive utility of these biomarkers in evaluating therapeutic efficacy.

Finally, given the heterogeneity of canine lymphoma, future trials should incorporate stratification based on lymphoma subtype, stage, and immunophenotype to better define the contexts in which rcIL-15 may offer the greatest benefit.

The findings of this study contribute to the integration of immunotherapy in veterinary oncology. The favorable safety profile of rcIL-15, combined with its potential immunomodulatory and tumor-suppressive effects, makes it a promising candidate for further development. Beyond lymphoma, IL-15-based therapies could have applications in other malignancies, where immune dysfunction plays a critical role. Moreover, the insights gained from this study may have translational relevance to human oncology, given the shared mechanisms of tumor immunity across species.

## 5 Conclusion

This study demonstrates that rcIL-15, when combined with conventional chemotherapy, has the potential to improve clinical outcomes, enhance tumor biomarker responses, and improve quality of life in dogs with lymphoma, all while maintaining a favorable safety profile. These findings provide preliminary support for the use of rcIL-15 as an adjunctive immunotherapeutic agent in canine lymphoma. Future studies with larger sample sizes, longer follow-up periods, and more rigorous stratification are essential to validate these results and to further explore the role of rcIL-15 in veterinary oncology.

## Data Availability

The original contributions presented in the study are included in the article/Supplementary material, further inquiries can be directed to the corresponding author.
