# Supplementary material for: Immunotherapeutic efficacy of recombinant canine IL-15 as an adjunct to chemotherapy in canine lymphoma
Source: Front Vet Sci. 2025 May 30;12:1596084. doi: 10.3389/fvets.2025.1596084 (PMC12162491; doi:10.3389/fvets.2025.1596084)
Supplement: Supplementary file 1 [file Table_1.docx]

**Supplementary Table 1. Owner-assessed quality of life in test and control groups in the lymphoma clinical trial**

| Questionnaires | Groups | | 0W | | 2W | | 4W | | 8W | | 12W | | *p*  (time) | *p*  (group) | *p*  (time* group) |
| --- | --- | --- | --- | --- | --- | --- | --- | --- | --- | --- | --- | --- | --- | --- | --- |
| **Joy of life** (0: Never, 1: Infrequently, 2: Sometimes, 3: Frequently, 4: Always) | | | | | | | | | | | | | | | |
| My pet has been playing a normal amount for him/her | | **Test** | | 2.17±1.58 | | 2.54±1.48 | | 3.00±0.91 | | 2.89±1.13 | | 3.00±1.08 | 0.021* | 0.143 | 0.729 |
|  |  | **Control** | | 2.89±1.33 | | 2.72±1.02 | | 3.37±0.96 | | 3.37±0.96 | | 3.21±1.03 |  |  |  |
| My pet has been responding to my presence | | **Test** | | 3.17±1.15 | | 3.35±1.14 | | 3.50±0.62 | | 3.61±0.61 | | 3.61±0.50 | 0.334 | 0.024* | 0.661 |
|  |  | **Control** | | 3.53±1.12 | | 3.28±0.83 | | 3.89±0.32 | | 3.79±0.42 | | 3.84±0.50 |  |  |  |
| My pet has been enjoying life | | **Test** | | 2.00±1.37 | | 2.43±1.39 | | 2.78±0.94 | | 2.83±1.04 | | 3.11±0.96 | 0.001* | 0.114 | 0.427 |
|  |  | **Control** | | 2.84±1.30 | | 2.72±0.67 | | 3.16±1.01 | | 3.16±1.07 | | 3.21±1.13 |  |  |  |
| My pet has been happy to see me when I get home | | **Test** | | 3.17±1.25 | | 3.38±1.19 | | 3.39±0.92 | | 3.44±0.70 | | 3.56±0.51 | 0.619 | 0.024* | 0.950 |
|  |  | **Control** | | 3.58±1.12 | | 3.61±0.61 | | 3.84±0.37 | | 3.79±0.42 | | 3.89±0.46 |  |  |  |
| **Mental status** (0: Strongly disagree, 1: Disagree, 2: Neutral, 3: Agree, 4: Strongly agree) | | | | | | | | | | | | | | | |
| My pet has had more good days than bad days | | **Test** | | 2.44±1.10 | | 2.68±1.18 | | 3.06±0.87 | | 2.94±0.80 | | 3.17±0.99 | 0.003* | 0.066 | 0.261 |
|  |  | **Control** | | 2.89±1.24 | | 3.00±0.69 | | 3.42±0.77 | | 3.58±0.61 | | 3.26±0.93 |  |  |  |
| My pet has been sleeping more than usual | | **Test** | | 2.39±1.34 | | 2.24±1.30 | | 1.50±0.86 | | 1.67±1.14 | | 1.67±1.03 | 0.001* | 0.834 | 0.440 |
|  |  | **Control** | | 2.11±1.29 | | 1.72±0.96 | | 1.47±1.47 | | 1.42±1.26 | | 2.00±1.45 |  |  |  |
| My pet has seemed depressed | | **Test** | | 1.89±1.23 | | 1.73±1.28 | | 1.00±0.97 | | 1.00±0.97 | | 1.06±1.06 | 0.621 | 0.000* | 0.551 |
|  |  | **Control** | | 1.58±1.35 | | 1.06±0.94 | | 1.00±1.25 | | 0.74±1.05 | | 1.16±1.01 |  |  |  |
| My pet has seemed anxious/stressed | | **Test** | | 1.67±1.09 | | 1.57±1.21 | | 0.94±1.00 | | 1.06±1.21 | | 0.94±1.21 | 0.001* | 0.873 | 0.143 |
|  |  | **Control** | | 1.47±1.35 | | 1.00±0.84 | | 1.16±1.42 | | 0.79±1.03 | | 1.32±1.20 |  |  |  |

*(Continue)*

| Questionnaires | | Groups | 0W | 2W | 4W | 8W | 12W | *p*  (time) | *p*  (group) | *p*  (time* group) |
| --- | --- | --- | --- | --- | --- | --- | --- | --- | --- | --- |
| **Pain** (0: Never, 1: Infrequently, 2: Sometimes, 3: Frequently, 4: Always) | | | | | | | | | | |
| My pet has been in pain | **Test** | | 0.83±1.20 | 0.89±1.33 | 0.50±0.86 | 0.56±1.29 | 0.39±0.98 | 0.115 | 0.920 | 0.461 |
|  | **Control** | | 0.95±1.47 | 0.61±0.85 | 0.63±1.12 | 0.53±1.02 | 0.63±0.96 |  |  |  |
| My pet has appeared restless | **Test** | | 0.56±0.98 | 0.65±1.09 | 0.28±0.75 | 0.39±0.92 | 0.44±0.78 | 0.090 | 0.683 | 0.833 |
|  | **Control** | | 0.74±1.20 | 0.33±0.59 | 0.37±0.68 | 0.53±0.96 | 0.42±0.77 |  |  |  |
| My pet has seemed painful in the tumor area | **Test** | | 0.50±1.15 | 0.41±1.04 | 0.39±0.85 | 0.50±1.20 | 0.50±1.15 | 0.437 | 0.714 | 0.505 |
|  | **Control** | | 0.32±0.95 | 0.33±0.69 | 0.11±0.32 | 0.21±0.54 | 0.32±0.67 |  |  |  |
| **Appetite/food intake** (0: Never, 1: Infrequently, 2: Sometimes, 3: Frequently, 4: Always) | | | | | | | | | | |
| My pet has been eating a normal amount | **Test** | | 2.83±1.51 | 2.95±1.37 | 3.28±0.83 | 3.33±0.59 | 3.17±1.10 | 0.521 | 0.253 | 0.949 |
|  | **Control** | | 3.05±1.27 | 3.11±0.76 | 3.42±1.02 | 3.26±1.37 | 3.32±1.20 |  |  |  |
| My pet has shown a capricious appetite | **Test** | | 1.17±1.25 | 1.03±1.32 | 1.06±1.30 | 1.50±1.34 | 0.89±1.02 | 0.660 | 0.040* | 0.360 |
|  | **Control** | | 0.89±1.41 | 1.17±0.99 | 0.68±1.25 | 0.53±1.02 | 0.74±1.28 |  |  |  |
| My pet has been eating his/her usual diet | **Test** | | 2.67±1.41 | 2.97±1.40 | 3.50±0.79 | 3.44±0.62 | 3.11±1.32 | 0.232 | 0.126 | 0.611 |
|  | **Control** | | 3.26±1.37 | 3.39±0.70 | 3.53±0.90 | 3.53±1.02 | 3.53±0.84 |  |  |  |
| My pet has shown difficulty in eating | **Test** | | 0.50±0.99 | 0.41±0.96 | 0.67±1.19 | 0.33±0.69 | 0.72±1.32 | 0.986 | 0.344 | 0.590 |
|  | **Control** | | 0.32±0.95 | 0.39±0.61 | 0.26±0.65 | 0.58±1.30 | 0.16±0.37 |  |  |  |
| Hygiene (0: Never, 1: Infrequently, 2: Sometimes, 3: Frequently, 4: Always) | | | | | | | | | | |
| My pet has been keeping himself/herself clean | **Test** | | 2.18±1.33 | 2.61±1.36 | 2.88±0.93 | 2.59±1.18 | 2.59±1.23 | 0.478 | 0.042* | 0.482 |
|  | **Control** | | 3.00±1.29 | 2.47±1.23 | 3.00±1.20 | 3.16±1.12 | 3.05±1.08 |  |  |  |
| My pet’s coat has been in good condition | **Test** | | 2.28±1.36 | 2.50±1.36 | 2.56±1.20 | 1.94±1.47 | 1.61±1.42 | 0.006* | 0.140 | 0.495 |
|  | **Control** | | 2.72±1.36 | 2.56±1.15 | 2.78±1.40 | 2.67±1.41 | 2.56±1.38 |  |  |  |

*(Continue)*

| Questionnaires | Groups | 0W | 2W | 4W | 8W | 12W | *p*  (time) | *p*  (group) | *p*  (time* group) |
| --- | --- | --- | --- | --- | --- | --- | --- | --- | --- |
| **Hydration status** (0: Never, 1: Infrequently, 2: Sometimes, 3: Frequently, 4: Always) | | | | | | | | | |
| My pet has been drinking a normal amount | **Test** | 3.12±1.11 | 3.36±1.05 | 3.47±0.62 | 3.12±0.86 | 3.24±0.75 | 0.685 | 0.031* | 0.707 |
|  | **Control** | 3.58±0.96 | 3.29±0.69 | 3.58±0.61 | 3.63±0.60 | 3.42±0.84 |  |  |  |
| My pet has been urinating normally | **Test** | 3.24±1.09 | 3.44±0.91 | 3.47±0.80 | 3.35±0.70 | 3.41±0.71 | 0.747 | 0.076 | 0.872 |
|  | **Control** | 3.63±0.68 | 3.29±0.85 | 3.68±0.58 | 3.58±0.96 | 3.53±0.84 |  |  |  |
| **Mobility** (0: Never, 1: Infrequently, 2: Sometimes, 3: Frequently, 4: Always) | | | | | | | | | |
| My pet has been moving around normally | **Test** | 2.67±1.28 | 2.95±1.31 | 3.50±0.62 | 3.22±0.81 | 3.22±0.94 | 0.271 | 0.176 | 0.363 |
|  | **Control** | 3.21±1.32 | 3.33±0.59 | 3.47±0.77 | 3.53±0.77 | 3.32±1.11 |  |  |  |
| My pet has shown difficulties in getting up | **Test** | 1.22±1.48 | 0.89±1.31 | 0.28±0.46 | 0.44±0.92 | 0.50±1.04 | 0.164 | 0.971 | 0.082 |
|  | **Control** | 0.58±1.07 | 0.44±0.70 | 0.68±1.34 | 0.84±1.38 | 0.89±1.41 |  |  |  |
| My pet’s activity level has been normal for him/her | **Test** | 2.06±1.26 | 2.46±1.35 | 3.50±0.71 | 2.94±1.11 | 3.22±1.06 | 0.000* | 0.385 | 0.012* |
|  | **Control** | 2.84±1.34 | 2.94±0.80 | 3.21±1.08 | 3.00±1.37 | 3.05±1.18 |  |  |  |

*(Continue)*

| Questionnaires | Groups | | 0W | 2W | 4W | 8W | 12W | *p*  (time) | *p*  (group) | *p*  (time* group) |
| --- | --- | --- | --- | --- | --- | --- | --- | --- | --- | --- |
| **Cardiovascular/respiratory system** (0: Never, 1: Infrequently, 2: Sometimes, 3: Frequently, 4: Always) | | | | | | | | | | |
| My pet’s breathing has been normal | | **Test** | 3.17±1.10 | 3.32±1.13 | 3.61±0.78 | 3.33±0.84 | 3.39±0.92 | 0.291 | 0.175 | 0.467 |
|  |  | **Control** | 3.47±1.17 | 3.22±0.81 | 3.68±0.58 | 3.74±0.56 | 3.47±0.84 |  |  |  |
| My pet has been getting tired easily | | **Test** | 1.67±1.28 | 1.68±1.36 | 0.94±0.87 | 0.83±0.86 | 1.00±1.08 | 0.007* | 0.837 | 0.649 |
|  |  | **Control** | 1.68±1.46 | 1.11±0.83 | 0.95±1.27 | 1.11±1.45 | 1.53±1.47 |  |  |  |
| My pet has shown coughing | | **Test** | 0.50±0.92 | 0.70±1.18 | 0.44±0.98 | 0.44±1.04 | 0.50±0.86 | 0.683 | 0.444 | 0.835 |
|  |  | **Control** | 0.89±1.37 | 0.67±1.08 | 0.68±1.00 | 0.63±1.12 | 0.74±1.28 |  |  |  |
| General health (0: Strongly disagree, 1: Disagree, 2: Neutral, 3: Agree, 4: Strongly agree) | | | | | | | | | | |
| My pet has generally been well | | **Test** | 2.61±1.20 | 2.89±1.15 | 3.33±0.77 | 3.28±0.75 | 3.17±0.99 | 0.016* | 0.192 | 0.410 |
|  |  | **Control** | 3.16±1.07 | 3.17±0.62 | 3.53±0.77 | 3.37±1.07 | 3.26±0.81 |  |  |  |
| Did the QoL of my pet drastically worsen after the  tumor diagnosis | | **Test** | 1.56±1.04 | 1.41±1.17 | 1.33±0.97 | 1.11±1.13 | 1.06±1.16 | 0.001* | 0.076 | 0.706 |
|  |  | **Control** | 1.26±1.28 | 1.50±1.15 | 0.84±1.07 | 0.53±1.07 | 0.79±1.13 |  |  |  |
| Did the QoL of my pet drastically worsen after  chemotherapy? | | **Test** | 1.18±0.95 | 1.26±1.19 | 1.18±0.73 | 0.94±1.09 | 0.88±0.86 | 0.157 | 0.367 | 0.689 |
|  |  | **Control** | 1.35±1.41 | 1.12±0.99 | 0.94±1.20 | 0.76±1.35 | 0.76±0.97 |  |  |  |
| My pet has been having a good QoL | | **Test** | 3.00±0.97 | 3.14±1.02 | 3.11±0.83 | 3.11±0.58 | 3.17±0.62 | 0.669 | 0.285 | 0.798 |
|  |  | **Control** | 3.28±1.07 | 3.11±0.68 | 3.22±0.88 | 3.22±0.88 | 3.44±0.78 |  |  |  |

Values are presented as mean ± standard deviation.
0W: Before administration of recombinant canine IL-15; 2W, 4W, 8W, 12W: 2, 4, 8, and 12 weeks after the first administration of recombinant canine IL-15, respectively.
*Statistical significance: *p* < 0.05.
